# Editorial: MSPP 34th scientific meeting: Pharmacological perspectives on natural products in drug discovery

**DOI:** 10.3389/fphar.2022.1049063

**Published:** 2022-10-11

**Authors:** Mohd. Farooq Shaikh, Sadhana Sathaye, Wan Amir Nizam Wan Ahmad

**Affiliations:** ^1^ Neuropharmacology Research Laboratory, Jeffrey Cheah School of Medicine and Health Sciences, Monash University Malaysia, Bandar Sunway, Selangor, Malaysia; ^2^ Department of Pharmaceutical Sciences and Technology, Institute of Chemical Technology, Mumbai, India; ^3^ Biomedicine Programme, School of Health Science, Universiti Sains Malaysia, Kelantan, Malaysia

**Keywords:** natural products, drug discovery, human diseases, disease modeling, drug testing

The aim of this Research Topic was to highlight advances in the field of translational research utilises natural products as an intervention strategy for various conditions, especially in the area of cancer, cardiovascular, neurological, and metabolic diseases. This Research Topic seeks to provide opportunities to share and exchange evidence-based practices and scientific endeavours concerning therapeutic natural products among physiologists, pharmacologists, physicians, general practitioners, research scientists and other health care professionals.

This Research Topic compiles 9 articles, including 4 reviews and 5 original research articles from prominent scientists in the field. This compilation of papers comprehensively covers a number of natural products in drug discovery and provides insights into molecular mechanisms of a number of conditions. The content of each article is summarised below.

An original research article by Liu et al. demonstrated the inhibitory effects of gypenoside L (Gyp L) and gypenoside LI (Gyp LI) in the proliferation and induced apoptosis in clear cell renal cell carcinoma (ccRCC) cells *via in vitro* studies. The authors also elucidated the mechanism of action of gypenoside treatment using an *in vivo* model *via* the downregulation of cPLA2 levels, reduction of arachidonic acid content and inhibition of tumour growth. Although further research is necessary, this study provided preliminary data to support that Gyp L and Gyp LI could be promising drugs in ccRCC treatment.

A comprehensive review by Fuloria et al. on the therapeutic potential of *Curcuma longa* Linn. about curcumin, its primary active constituent. Curcumin is universally known for its therapeutic effects in multiple disorders, but the same cannot be said for *C. longa*. The review provided extensive information about *C. longa* and discussed the knowledge gap in traditional and scientific evidence about *C. longa* and curcumin. Despite all the promising evidence about C. longa, the authors established that there is still inadequate supportive evidence, especially from clinical studies on the adjunct use of *C. longa* and curcumin. This calls for more clinical and pre-clinical studies on *C. longa* and curcumin.

*Channa striatus* (CS) is traditionally consumed by Malaysians, believed to promote wound healing and alleviate inflammation. Lee et al. presented a study to investigate the anticonvulsive potential of CS extract on neuroinflammation-induced seizures using zebrafish. The authors developed a zebrafish model of neuroinflammation using cerebroventricular microinjection of lipopolysaccharide. Zebrafish behaviour and swimming pattern were analysed together with gene expression to demonstrate the pharmacological property of CS. CS extract treatment exhibited some anticonvulsive and anti-inflammatory activity, which provides the basis for future research to discover new therapeutics for epileptic seizures potentially.

A review by Fuloria et al. discussed the 590 biologically active meroterpenoids from various sources such as fungus, plants and marine organisms. Meroterpenoids have been reported to have pharmacological properties such as anti-cholinesterase, COX-2 inhibitory, anti-leishmanial, anti-diabetic and many more.

In an original research article, George et al. demonstrated that caloric vestibular stimulation (CVS) is a safe and straightforward neuroprotective treatment against stress and qualifies as a non-invasive therapy for overcoming motor symptoms associated with Chronic Mild Stress. CVS was found to improve behavioural and immunohistochemical modifications, which are indicators of neurodegeneration. This study paves the way for more research to study the therapeutic potential of CVS as a neuroprotectant in stress-related disorders.

Rahman et al. presented an original research article which reported the anticancer effects of hydroxychavicol (HC) and epigallocatechin-3-gallate (EGCG) against cancer cells. Their individual therapeutic properties have been reported before. Therefore Rahman et al., hypothesized that combining these two compounds may enhance the therapeutic activity. It was proposed that HC + ECGC halted glioma cell proliferation and exerted apoptotic effects on glioma cells. The mechanisms of action were elucidated in the article as well. More research needs to be done to investigate this promising treatment against glioma cancer.

A narrative review by Hossain et al. summarises colorectal cancer (CRC) prevention and treatment using herbs and spices. Due to the protective effects of herbs and spices, they have been deemed a safer natural alternative which can be used as an adjuvant therapy against CRC. The six herbs and spices selected for the review were ginger, turmeric, garlic, fenugreek, sesame, and flaxseed. The authors provided evidence to show the potential roles of these herbs and spices in preventing and reducing CRC severity. The authors also identified several challenges in incorporating intestinal microbiota into the treatment of CRC and proposed several steps for the appropriate management of CRC.

The study by Xu et al. investigated the effects and underlying mechanisms of methyl 6-O-cinnamoyl-α-d-glucopyranoside (MCGP) on acute liver injury caused by acetaminophen (APAP) or carbon tetrachloride (CCl_4_). MCGP is a modified compound from cinnamic acid, which has significant antioxidant, anti-inflammatory and anti-diabetic effects. The protective effects of MCGP in APAP-/CCl4-intoxicated acute liver injury were identified for the first time. The authors suggested the mechanism of action of MCGP, which paves the way for more research.

A review article by Lambuk et al. outlined the neuroprotective potential of brain-derived neurotrophic factor (BDNF) in Glaucoma. The authors highlighted the possibility of using BDNF as a biomarker in neurodegenerative diseases and future strategies. The review featured the various sources of BDNF, its transport mechanisms within neurons and its extensive functions. The highlighted therapeutic benefits help physicians to remain updated on recent discoveries.

The editorial team is grateful of all the authors and review editors for their contributions to the special Research Topic. We are confident that these diverse, interesting and important papers will guide researchers in their future research and will kindle advanced discussions in translational evidence based complementary and alternative medicine research.

